# Impairment of Intestinal Barrier Function Induced by Early Weaning *via* Autophagy and Apoptosis Associated With Gut Microbiome and Metabolites

**DOI:** 10.3389/fimmu.2021.804870

**Published:** 2021-12-15

**Authors:** Wenjie Tang, Jingliang Liu, Yanfei Ma, Yusen Wei, Jianxin Liu, Haifeng Wang

**Affiliations:** College of Animal Science, Zhejiang University, The Key Laboratory of Molecular Animal Nutrition, Ministry of Education, Hangzhou, China

**Keywords:** weaned piglet, intestinal barrier, apoptosis, autophagy, gut microbiota, metabolome

## Abstract

Early weaning piglet is frequently accompanied by severe enteric inflammatory responses and microbiota dysbiosis. The links between the gut microbiome and the etiology of gut inflammation are not fully understood. The study is aimed to investigate the potential molecular mechanisms mediating inflammatory reactivity following early weaning, and to find whether these changes are correlated with gut microbiota and metabolite signatures by comparison between suckling piglets (SPs) and weaning piglets (WPs). Histopathology analysis showed a severe inflammatory response and the disruption of epithelial barrier function. Early weaning resulted in reduced autophagy indicated as the suppression of autophagic flux, whereas induced the TLR4/P38MAPK/IL-1β-mediated apoptotic pathway, as well as activation of the IL-1β precursor. The alpha-diversity and microbial composition were changed in WPs, such as the decreased abundances of *Bifidobacterium*, *Bacteroides*, *Bacillus*, *Lactobacillus*, and *Ruminococcus*. Microbial co-concurrence analysis revealed that early weaning significantly decreased network complexity, including network size, degree, average clustering coefficient and number of keystone species, as compared with the SP group. Differentially abundant metabolites were mainly associated with amino acid and purine metabolism. Strong correlations were detected between discrepant microbial taxa and multiple inflammatory parameters. In conclusion, we found that dysregulations of autophagy and apoptosis pathway were involved in colon inflammation during weaned period, which may result from gut microbiota dysbiosis. This study may provide possible intervention modalities for preventing or treating post-weaning infections through maintaining gut microbial ecosystem integrity.

## Introduction

Early weaning is a widely used strategy for improving sow breeding efficiency in the swine breeding industry. However, early weaning exposes piglets to multiple environmental stresses, such as separation from the sow, a switch in fibrous feed, handling and litter mixing. These experiences result in serious gut health concerns and, eventually, economic losses (1, 2). The circumstances of weaning transition generally induced gastrointestinal infections *via* mucosal surfaces, which attributes to around 17% losses of piglets born in Europe (3). Accumulating evidence has indicated that mammalian offspring are highly susceptible to enteric diseases during the transition of the early weaning (4, 5). Due to the immature gastrointestinal (GI) tract, young animals experience large changes in intestinal morphology, architecture, and physiological functions under weaning stressors (6, 7). The intestinal immune system and barrier function of the pre-weaning piglet have not specialized and expanded completely (3). All these external and internal factors inevitably affect the intestinal development of weaned piglets (WPs) and can lead to inflammation. It is necessary to explore the mechanism how early weaning induces intestinal inflammation.

Autophagy and apoptosis represent distinct adaptive forms of programmed cell death, that are essential for maintaining the complex interplay between the host and gut microbes (8, 9). Excessive apoptosis induced by extracellular stimuli inevitably results in aberrant cell death and inflammation. Autophagy, meanwhile, is a highly conserved mechanism that plays dual roles in routine cell turnover and cellular homeostasis (10). Recent studies have implicated that dysregulated apoptosis and autophagy in intestinal tissues following gut microbiota dysbiosis can lead to bacterial dissemination and an overactive inflammatory response (11, 12). However, it is not clear how these two processes promote specific immunopathological characteristics in the intestinal epithelium of piglets under early weaning stress. Studies have shown that Toll-like receptor 4 (TLR4) is a critical mediator of inflammation and adaptive immunity (13). Mitogen-activated protein kinase (MAPK), a downstream target of TLR4, plays a key role in the progression of mucosal inflammation through the stimulation of an apoptotic cascade (14). These observations imply that the TLR4/P38MAPK signaling pathway may participate in the progression of gut inflammation induced by disturbances in the gut microbiota. However, early weaning-induced regulation of TLR4/P38MAPK or autophagy signaling pathway associated with intestinal homeostasis have not been fully characterized.

The gut microbiota plays a crucial role in the health of the host by producing components and metabolites essential for many biological processes, such as immune regulation, the maintenance of enterocyte function, and the stabilization of the microecosystem (15, 16). The intestinal microbiota of pig comprises a vast and complex community exhibiting dynamic taxonomic richness and diversity that fluctuate over time and along the longitudinal axis of the GI tract (17). Gut microbiota assembly is initiated at birth and develops toward a breastfeeding-oriented microbiome during lactation (18). An abrupt shift from maternal milk to complex solid feed is deemed to result in the remodeling of the gut microbiota structure in piglets. Thus, the examination of the composition of the gut microbiota is important for explaining enteric diseases in WPs. The ecological roles of individual microbial members possess uneven importance within microecosystems (19). The remodeling of the intestinal microbiota structure by weaning transition strongly affects co-occurrence patterns of microorganism. The altered microorganism co-occurrence patterns may further have an impact on the gut health of piglets. Computational-derived co-occurrence network hubs are conducive to determining whether the core ecological modules formed by microorganisms would change within different responsiveness to intervention (20).

The dysbiosis of the intestinal flora frequently alters metabolomic profiles in the GI content, which is thought to be related to modifications in microbe-derived gut metabolites (21). The microbiota-derived metabolites are key intermediates in host-microbiota interactions and influences a wide variety of physiological functions (16, 22). During the weaning transition, solid feed consumed by piglets provides heterogeneous fermentation substrates and prolonged transit time in the GI tract (23). These changes are expected to affect microbial metabolic activity. However, how early weaning influences the gut microbiota and the metabolome of piglets remains incompletely understood. Here, we speculated that the gut metabolome of piglets may undergo profound alterations at the weaning transition period. The exploration of the potential role of the intestinal microbiota and associated metabolomes would help to find the determinants of gut inflammatory disease in piglets.

In this study, the genetic background, age, and feeding environment of the animal were strictly controlled (24). We hypothesized that early weaning induced the colon inflammatory response through dysregulating the autophagy and apoptosis signaling pathways, and alternations of gut microbiota and metabolome are associated with the dysregulation of these two signaling pathways.

## Materials and Methods

### Animal Care and Experimental Design

All procedures involving animals were performed in full compliance with the “Regulation for the Use of Experimental Animals” of Zhejiang Province, China. This research was specifically approved by the Animal Care and Use Committee of Zhejiang University (Ethics Code Permit ZJU20170529). Twelve piglets (Landrace × Yorkshire) were selected from three litters. At age of 21 days, piglets (body weight, 6.95 ± 0.73 kg) from the same litter were randomly allocated to two treatments. The two groups were as follows: suckling piglet group (SP), were continued to be breastfed by sows; and weaning piglet group (WP), were separated from the sows and had *ad libitum* access to water and feed (*n* = 6/group). At age of 25 days, all piglets were euthanized *via* an intravenous injection of sodium pentobarbital solution (25 mg/kg body weight). Colonic content and tissue were harvested and lyophilized. Tissues adjacent to the colon were stored in 2.5% glutaraldehyde for evaluation by transmission electron microscopy (TEM). Colonic tissue was fixed in 4% buffered formalin for paraffin embedding. Colonic mucosa was collected with a microscope slide and immediately stored at −80°C until use.

### Morphology, Histological Score, and TUNEL Labeling

Specimens of the colon were dehydrated with ethanol, cleared with xylene, embedded in paraffin, and cut into 4-μm-thick serial sections. After deparaffinization, the sections were stained with hematoxylin and eosin (H&E) for morphometric analysis. Crypt depth was scored by capturing image of colon tissue in H&E staining slides at 10× using a Bx63-Olympus microscope (Olympus, USA). The images were analyzed using the pen tool form Image J software (ImageJ, NIH, USA). For each sample, at least 20 intact and well-oriented crypts were measured as distance from the apical side to the basal side of crypt. The crypt depth data was shown as mean value. Intestinal acidic and neutral mucin content was examined using periodic acid-Schiff-alcian blue (PAS-AB; Sigma Aldrich) staining. Images were acquired using a scanner (Leica Aperio CS2, San Diego, CA, USA). Morphology was analyzed using the Aperio XT system (San Diego, CA, USA). To assess intestinal inflammation severity, colonic histopathological scoring of H&E-stained sections (100× magnification) were performed. Two blinded histopathological scoring methods were carried out using the following four criteria: 1) the extent of inflammation, 2) bleeding, 3) crypt hyperplasia, and 4) crypt density loss (**Supplementary Table 1**). Each criterion score ranged from 0 to 3 and the sum of the scores for the four criteria were calculated. A TUNEL Apoptosis Assay Kit (Roche, Sweden) was used to visualize the extent of cell apoptosis in paraffin-embedded colonic tissue. Images were captured using an Olympus VS200 digital slide scanner (Japan) to judge the difference in TUNEL-positive cell counts between the two groups. The nuclei of apoptotic cells were identified by the presence of green fluorescence.

### Detection of Cytokines in Serum and Colon Mucosa

Venous blood sample was drawn from SPs and WPs into sterile collection tubes. Blood was centrifuged at 3,000 rpm for 10 min at 4°C to collect serum. The colonic mucosa (100 mg) was added to 1 mL of pre-cooled phosphate-buffered saline (PBS) and ground using a tissue grinder (Tissuelyser-24, JingXin, Shanghai, China). The supernatant of the colonic mucosa homogenate was extracted after centrifugation at 3,000 rpm for 12 min at 4°C. The concentrations of IL-1β, IL-6, IL-10, IFN-γ, and TNF-α in the serum and tissue supernatants were detected using porcine ELISA assay kits (R&D Systems, Minneapolis, MN, USA) according to the manufacturer’s protocols. To calculate the concentration of each cytokine, the optical density was measured using a standard microplate reader (Phenix Research Products, Hayward, CA).

### Immunofluorescence (IF) and Immunohistochemistry (IHC)

Colon sections were kept in neutral formalin buffer for 12 h and then embedded in paraffin and sliced into 4-μm-thick sections. The sections were subsequently dewaxed and rehydrated. For IF staining, the sections were processed with antigen retrieval, permeabilization and blocking. The sections were incubated first with a primary antibody (rabbit anti-MUC2, Biorbyt Ltd, orb372331, 1:400) for 8 h at 4°C. Then, the sections were washed and incubated with a fluorescence-labeled secondary antibody (Alexa Fluor 488-conjugated anti-rabbit IgG, Abcam, #ab150077, 1:600) for 2 h. Nuclei were counterstained with 4ʹ,6-diamidino-2-phenylindole (DAPI) (Cell Signaling Technology, #8961, 150 ng/mL) for 20 min. For IHC, the sections were subjected to antigen retrieval by incubating in AR9 buffer (pH 6.0, PerkinElmer) at 96°C for 20 min. Then, 3% H_2_O_2_ in methanol was used to quench endogenous peroxidase activity on samples. The sections were followed by incubation with primary antibody (rabbit anti-LC3A/B, Cell Signaling Technology, #12741, 1:450) for 8 h at 4°C. A DAB HRO substrate kit was used for visualization following the manufacturer’s instructions. Images were captured using a Bx63-Olympus microscope (Olympus, USA) and analyzed using ImageJ software.

### TEM

Small pieces (1.5–2.5 mm^3^) of colonic specimens were immersed in a mixture of 2% glutaraldehyde and 0.1 M cacodylate buffer (pH 7.4) until needed. The specimens were washed twice with PBS, incubated with 1% osmium tetroxide in PBS at 4°C for 40 min, and then washed again twice with PBS. The specimens were dehydrated in a series of increasing ethanol concentrations (20%, 40%, 60%, 80%, and 100% *v*/*v*) with 15 min each step. After fixation in pure Spurr resin for 12 h, the specimens were embedded in capsules containing embedding medium and polymerized at 65°C for 4 h. The sections were stained with 0.5% uranyl acetate and alkaline lead citrate (AC20, Leica) for 15 min. Finally, the sections were imaged by TEM using a Model H-7650 microscope.

### Immunoblotting

Total protein was extracted from colonic tissue and lysed by lysis buffer (Solarbio, Beijing, China). The protein content of the supernatants or serum was concentrated using StrataClean resin (Agilent, CA, USA). All protein samples were prepared according to the manufacturer’s instructions. The protein samples were separated by 12% SDS–PAGE and transferred to PVDF membranes. The membranes were blocked in TBST buffer containing 3% BSA (*w*/*v*) for 60 min at room temperature. Then, the membrane was incubated first with primary antibodies (diluted in TBST buffer containing 1% BSA [*w*/*v*]) targeting primary antibodies overnight at 4°C. They were followed treatment with the appropriate HRP-conjugated secondary antibodies (anti-mouse IgG and anti-rabbit IgG; all 1:4,000) (EMD Millipore, MA, USA) diluted in TBST buffer containing 1% BSA (*w*/*v*) for 1 h at room temperature. Finally, the protein bands were developed using SuperSignal Chemiluminescent Substrate (Thermo Fisher Scientific, MA, USA). Primary antibody details were listed in **Supplementary Table 2**.

### 16S rRNA Library Construction and Sequencing

Bacterial genomic DNA was extracted from samples of colonic content using the Omega E.Z.N.A. Stool DNA Kit following the manufacturer’s protocol. DNA concentration and purity were detected using both a NanoDrop One (Thermo Scientific) spectrophotometer and a Qubit 2.0 Fluorometer (Thermo Scientific). Libraries were constructed and sequenced at the Realbio Genomics Institute (Shanghai, China). Briefly, the V3–V4 region of the 16S rRNA gene was PCR-amplified using the primer pair 341F (5′-CCTACGGGNGGCWGCAG-3′) and 806R (5′-GACTACHVGGGTATCTAATCC-3′). The amplicons were converted to sequencing libraries using the Illumina TruSeq DNA PCR-Free Library Preparation Kit (Illumina, USA). The libraries were sequenced on the MiSeq/HiSeq platform (Illumina, Inc., San Diego, CA, USA) to generate paired-end 500/250 bp reads. Demultiplexed fastq files were generated from the raw data using bcl2fast (v2.20.0.422).

### Bioinformatics Analysis of the Sequencing Data

Raw sequencing data from the 16S rRNA V3–V4 hypervariable region were submitted to the following three steps: i) Raw reads were first filtered using Trimmomatic v0.33 and then Cutadapt (version 1.9.1) to remove standard primer sequences to generate high-quality reads; ii) high-quality clean reads were assembled using FLASH v1.2.7; iii) sequences were denoised using dada2 (25) in QIIME2-2020.6 (26) to generate non-chimeric reads. Data quality was estimated based on read length and counts at each stage (**Supplementary Table 2**). An amplicon sequence variant (ASV) table was generated using the standard dada2 workflow. Based on the ASV table, common and unique features between the two groups were visualized using a Venn diagram (27). Taxonomic classification of the ASVs was achieved by Bayesian classifier and BLAST using GreenGene as the reference database. QIIME2 was applied to calculate the abundance of each species and the distribution at each taxonomic level. Alpha diversity was evaluated using species richness and the corresponding significance was determined using the Wilcoxon rank sum test. Beta diversity was estimated using principal coordinate analysis (PCoA) on a matrix of Bray–Curtis distances using QIIME2 software. The statistical significance of the PCoA results was determined using PERMANOVA. According to the NCBI taxonomy database for existing microbial species, MEGAN software was applied to combine species abundance information obtained by sequencing into a taxonomic tree (28). Significant differences in relative abundance between the groups were determined using the Wilcoxon rank sum test and corrected *P*-values (false discovery rate [FDR]). Predictive metabolic functional analysis was performed using the PICRUSt2 plugin for QIIME2 (29). Pathways were assigned based on the KEGG Orthology database while DESeq2 was used for differential abundance testing.

### Co-Occurrence Network Diagram

Spearman’s rank correlation analysis was used to analyze the differences in the abundance of all species among samples. Data with a correlation coefficient |*ρ*-value| >0.5 and a *P*-value <0.05 were selected to construct a co-occurrence network. Subnetworks were isolated from the whole network using the following filters: ASVs comprising >0.01% of the total relative abundance and taxa present in >30% of the samples. The network diagram was visualized using a customized Organic layout algorithm in Cytoscape (v3.6.0). For both groups, the five modules were obtained using the vertex and edge betweenness centrality functions in the igraph R package (19). Node-level topological properties were calculated using Gephi (v0.9.2), the properties include degree, clustering coefficient, closeness centrality, betweeness centrality, Zi (within-module connectivity) and Pi (among-module connectivity) (30, 31). Statistically significant differences in node-level attributes were determined by non-parametric Mann–Whitney U tests.

### Metabolomics Profiling

A total of 50 ± 1 mg of colonic content was added to 500 μL of a pre-cooled methanol/chloroform extraction mixture in a 3:1 (*v*/*v*) ratio. The samples were mixed with 10 μL of internal standard (Adonitol, 0.5 mg/mL stock). Then, the samples were vortexed and homogenized using a ball mill for 4 min at 50 Hz, and followed by ultrasonication for 5 min in ice-cold water. After centrifugation at 4°C for 15 min at 12,000 rpm, 200 μL of the supernatant was transferred to a fresh tube. To prepare the quality control (QC) sample, 18 μL of each sample were combined. After evaporation in a vacuum concentrator, 80 μL of methoxyamine hydrochloride (20 mg/mL in pyridine) was added followed by incubation at 80°C for 30 min and derivatization by 1 mL of BSTFA regent (1% TMCS, *v*/*v*) at 70°C for 1.5 h. Fatty acid methyl esters (FAMEs; 5 μL in chloroform) were added to the QC sample. Gas chromatography time-of-flight mass spectrometry (GC–TOF–MS) was performed using an Agilent 7890 gas chromatograph coupled with a time-of-flight mass spectrometer (DB-5MS capillary column). A 1 μL aliquot of sample was injected in splitless mode. Helium was used as the carrier gas, the front inlet purge flow was 3 mL/min. The gas flow rate through the column was 1 mL/min. The initial temperature was kept at 50°C for 1 min, raised to 310°C at a rate of 10°C/min, and kept for 8 min at 310°C. The injection, transfer line, and ion source temperatures were set at 250, 280, and 280°C, respectively. The mass spectrometry data were obtained in full-scan mode within the mass range of 50 to 500 *m*/*z* at a rate of 15.5 spectra per second after a solvent delay of 6.25 min.

### Metabolomics Data Analysis

Metabolomics data analysis was performed as previously described (32) with some modifications. Briefly, raw data were processed using Chroma TOF4.3x software (32). The LECO-Fiehn Rtx5 database was used for metabolite identification. Finally, peaks detected in less than half of the QC samples or those with a relative standard deviation (RSD) >30% in the QC samples were removed (33). The final dataset was imported into SIMCA15.0.2 (Sartorius Stedim Data Analytics AB, Umea, Sweden) for principal component analysis (PCA) and orthogonal partial least squares discriminant analysis (OPLS-DA). The variable importance in projection (VIP) score of the first principal component in the OPLS-DA was also calculated, which summarizes the importance of each variable to the model. Metabolites with a VIP score >1 and a Student’s *t*-test *P-*value <0.05 were determined to display significantly different abundance. The KEGG and MetaboAnalyst (http://www.metaboanalyst.ca) databases were referenced for pathway enrichment analysis. A correlation analysis between genera and metabolites was undertaken using Spearman’s correlation in R (Hmisc package, v3.4.3) and visualized using a heatmap.

### Statistical Analysis

The statistical significance of differences was assessed using the Student’s unpaired *t*-test, the Wilcoxon rank sum test, Mann–Whitney non-parametric tests, or PERMANOVA where appropriate. The results are presented as means ± SEM. Data were analyzed in SPSS (v2.1.0; IBM, USA) or R (for 16S rRNA sequencing data and the metabolomics profile). A *P-*value <0.05 was considered statistically significant and a 0.05 ≤ *P-*value <0.10 was considered a tendency.

## Results

### Comparison of the Colonic Morphology and Barrier Function Between WPs and SPs

**Figure 1:**
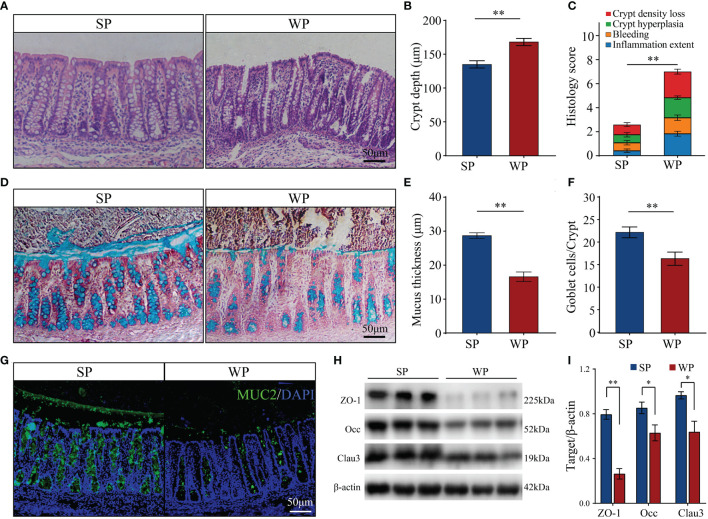
Effects of early weaning on colonic morphology and mucosal barrier function. **(A)** Representative images of hematoxylin and eosin-stained colon sections from SP and WP groups (scale bars, 50 μm). **(B)** Statistical analysis of crypt depth. **(C)** Stacked bar graph showing the total histology scores and individual histological criteria score differences between the two groups. **(D)** Representative images of periodic acid-Schiff-alcian blue (PAS-AB)-stained colonic sections from the SP and WP groups (scale bars, 50 μm). **(E)** Mucin layer width in the colon of SPs and WPs was quantified from images of AB staining. **(F)** Statistical analysis of the numbers of mucus-producing goblet cells. **(G)** Immunofluorescence staining for the goblet cell marker MUC2 (green). Scale bars, 50 μm. **(H, I)** Western blotting results for ZO-1, occludin, and claudin-3 expression in the colon. All data are presented as means ± SEM, Student’s *t*-test, *P < 0.05, **P < 0.01; *n* = 6. SP, suckling piglet; WP, weaning piglets.

Colonic morphological examination showed that, compared with the SP group, WPs displayed a loosely adherent intestinal epithelial layer structure and less intact surface, as well as erosion and crypt abscesses (**Figure 1A**). The WP group was characterized by a significantly greater crypt depth compared with that of the SP group (**Figure 1B**). In addition, histopathological evaluation revealed that the WP group was characterized by significantly greater crypt density loss, degree of bleeding and inflammatory cell infiltration compared with the SP group (**Figure 1C**). PAS-AB staining analysis showed that the thickness of the mucosal layer and the number of secretory goblet cells were significantly decreased in the WP group compared with those in the SP group (**Figures 1D–F**). IF staining for MUC2 further confirmed the reduced abundance of mucin-producing goblet cells in the WP group compared with that in the SP group (**Figure 1G**). The protein expression of zonula occludens-1 (ZO-1), occludin, and claudin 3 were significantly lower in the WP group than in the SP group (**Figures 1H, I** and **Supplementary Figure 1**).

### Early Weaning Induced Intestinal Impairment *via* Inhibiting Autophagy and Activating the Apoptotic Signaling Pathway

**Figure 2:**
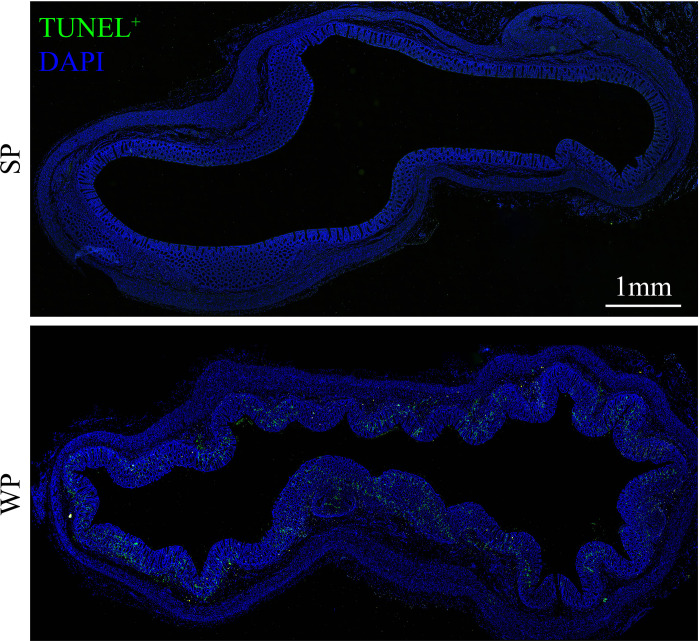
TUNEL staining in colonic sections from SPs and WPs. The nuclei of apoptotic cells are positive for TUNEL staining (green). Scale bars, 1 mm.

**Figure 3:**
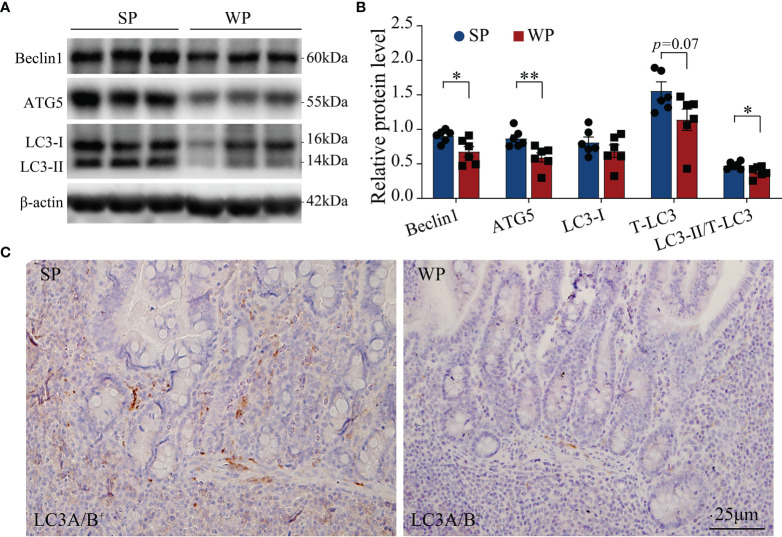
Autophagy-related signaling was suppressed in WPs relative to that in SPs. **(A, B)** The expression of autophagy-related proteins in colonic tissue was determined by immunoblotting. **(C)** Representative images of immunohistochemistry for LC3A/B (*n* = 6). Scale bars, 25 μm. The data are presented as the means ± SEM, Student’s *t*-test, *P < 0.05, **P < 0.01; *n* = 6.

The TUNEL assay results showed the presence of a significantly greater number of apoptotic cells in the colonic submucosa of WPs compared with that in the SP group (**Figure 2** and **Supplementary Figure 2**). The accumulation of apoptotic cells may be linked with modulating autophagy and apoptotic signal pathway. Therefore, we first examined the expression levels of autophagy-related proteins. The expression levels of beclin-1 and ATG5 were significantly decreased in the WP group compared with those of the SP group, which was in accordance with the reduced expression of microtubule-associated protein light chain 3 (LC3)-I and a decrease trend in expression of LC3-II (**Figures 3A, B** and **Supplementary Figure 3**). Similarly, IHC staining indicated that the number of LC3 puncta was lower in the WP group than in the SP group (**Figure 3C**).

**Figure 4:**
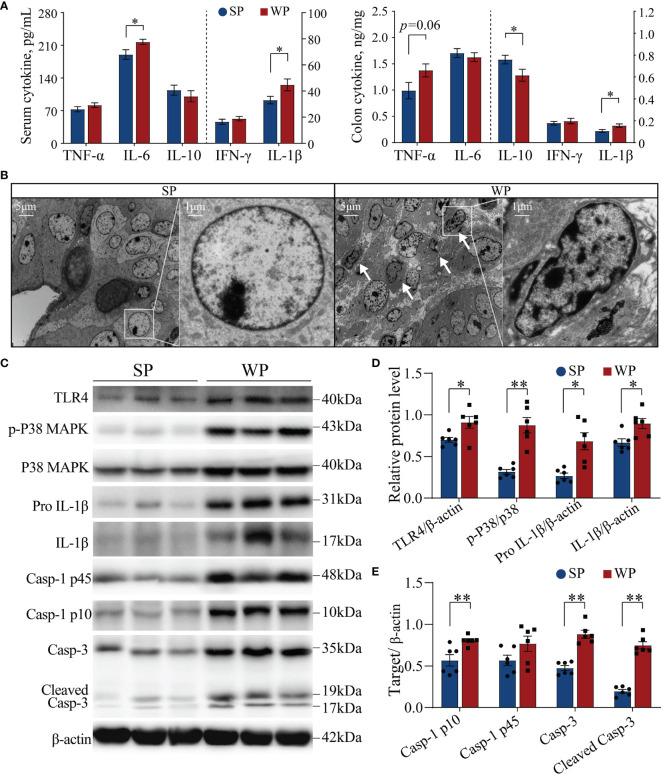
The TLR4/P38MAPK/IL-1β-mediated apoptosis signaling pathway was activated in WPs but not in SPs. **(A)** The levels of various cytokines in serum and colon tissue measured by ELISA. **(B)** Representative transmission electron micrographs of colonic crypts from the SP and WP groups. The low magnification image (scale bar, 5 μm; ×3,000 magnification) demonstrates abundant degeneration and the presence of necrotic cells scattered at the bottom of crypts in the WP group. The higher magnification image (scale bar, 1 μm; ×10,000 magnification) shows a typical apoptosis event characterized by fragmented nuclei and leaking of the nuclear mass (arrow). **(C–E)** The expression of components of the TLR4/P38MAPK/IL-1β signaling pathway and caspase-related proteins in colon tissue were determined by immunoblotting. All data are presented as the means ± SEM, Student’s *t*-test, *P < 0.05, **P < 0.01; *n* = 6.

The WP group had significantly higher serum IL-6 and IL-1β concentration, and had significantly higher colonic IL-1β and lower IL-10 content than the SP group (**Figure 4A**). Meanwhile, the colonic TNF-α content had a trend increase in the WPs than the SP group (**Figure 4A**). Ultrastructure analysis showed that a large number of damaged cells were detached and scattered at the bottom of the colon crypts in the WP group, which was accompanied by nuclear fragmentation as a hallmark of cell apoptosis (**Figure 4B** and **Supplementary Figure 4**). Accordingly, we further assessed the status of the TLR4/P38MAPK-mediated apoptotic signaling pathway, which functions upstream of IL-1β. Western blotting results showed that the protein levels of TLR4, pro IL-1β and IL-β were significantly higher in the WP group than in the SP group (**Figures 4C, D** and **Supplementary Figure 5**). Additionally, the ratio of p-P38MAPK to MAPK was significantly increased in the WP group than in the SP group (**Figures 4C, D** and **Supplementary Figure 5**). Moreover, the concentrations of caspase-1 p10, caspase-3, and cleaved (activated) caspase-3 were also significantly higher in the WP group than in the SP group (**Figures 4C, E** and **Supplementary Figure 5**), which indicated the greater rate of IL-1β conversion existed in the WP group than in the SP group.

### The Microbial Community Structure and the Co-Existence Pattern Differed Between the WP and SP Groups

**Figure 5:**
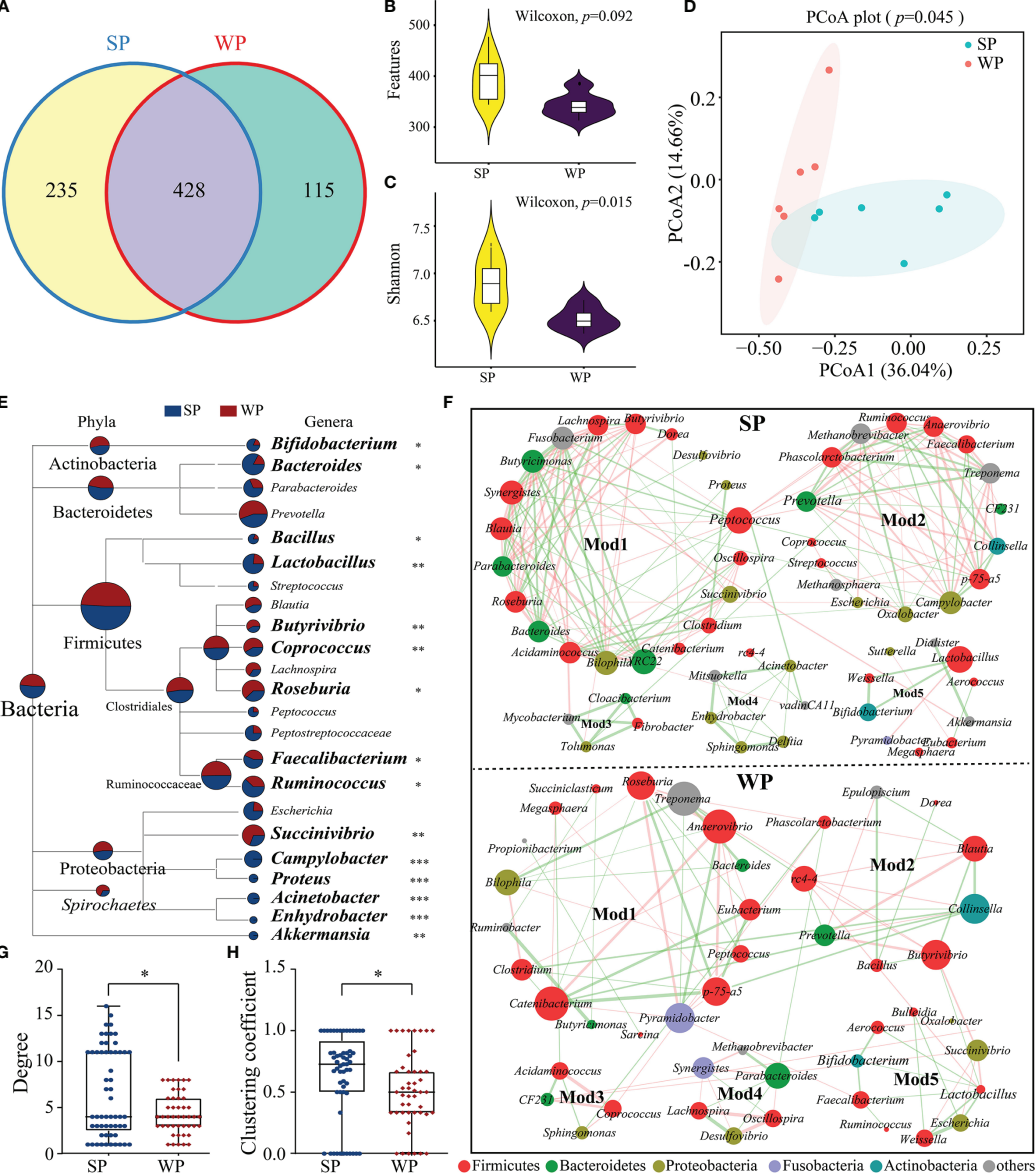
Differences in gut microbiota structure and co-occurrence patterns between the SP and WP groups. **(A)** Venn diagram displaying the numbers of identified amplicon sequence variants (ASVs) in the SP and WP groups. **(B, C)** Differences in alpha diversity in the gut bacteria were assessed based on features and the Shannon index. **(D)** Principal coordinate analysis (PCoA) plots based on the Bray–Curtis distances identified distinct clusters between the SP and WP groups. **(E)** MEGAN taxonomic tree emphasized the evolutionary relationship and differences in abundance of microorganisms in the SP and WP groups. Relative abundance is displayed in a colored pie-chart; the size of the pie-chart indicates relative sequence abundance. **(F)** Microbial co-occurrence patterns in the colon ecosystems of the SP and WP groups were visualized using network diagrams. Co-occurrence relationships with a strong Spearman’s correlation coefficient (|ρ value| >0.5 and *P* < 0.05) were selected and formed five modules in both groups; the size of each node is proportional to the degree of correlation. Phylum information for each node is indicated by color. The edges represent the correlation between the two genera, the thickness of the edge represents the strength of the correlation, orange represents a positive correlation, and green a negative correlation. **(G, H)** Topological properties (degree and clustering coefficient) of co-occurrence networks for bacterial communities in the SP and WP groups. All data are presented as means ± SEM. Wilcoxon rank sum test for **(B, C, F)**; PERMANOVA for **(D)**; and the Mann–Whitney non-parametric test for **(G, H)**. *P < 0.05, **P < 0.01, ***P < 0.001; *n* = 6.

The number of denoised clean reads (average of 31,439) was determined for each sample (**Supplementary Table 3**). Rarefaction curves for the ASVs indicated that all the samples achieved a high sampling coverage (>99%) (**Supplementary Figure 6** and **Supplementary Table 3**). These raw data analyses demonstrated that the sequencing depth was sufficient for analyzing the colonic microbiota. Next, we compared the results of 16S rRNA gene sequencing between the SP and WP groups. Common and unique ASV features between the groups were visualized by Venn diagram (**Figure 5A**), from which 235 and 115 unique ASVs were classified in the SP and WP group, respectively. As shown in **Supplementary Table 4**, differences in α-diversity were found between the two groups indicated as common and unique ASV features and the Shannon, Simpson, and Chao1 indices. The WP group had a lower trend inASVs than the SP group (Wilcoxon rank sum test, *P* = 0.092) (**Figure 5B**). The Shannon index was lower in the WP group than in the SP group (Wilcoxon rank sum test, *P* = 0.015) (**Figure 5C**). We found a clear separation between the two groups, which suggests significantly distinct in bacterial community composition between the SP and WP group, respectively (PERMANOVA, *P* = 0.045) (**Figure 5D**).

Relative abundance values were used to calculate the biomass for each species and generate a distribution histogram at each taxonomic level. At the phylum level, bacterial communities in the piglet colon were dominated by *Firmicutes* (61.87%–63.57%), *Bacteroidetes* (22.76%–23.47%), and *Proteobacteria* (4.18%–4.93%) (**Supplementary Figure 7A**). No significant differences were found in read numbers or relative abundance between the SP and the WP groups (**Supplementary Figures 7B, C**). At the genus level, we identified a total of 83 genera from the core bacterial community and combined relative abundance information to taxonomic tree (provided by NCBI) (**Figure 5E**). The results showed a comprehensive framework of the taxonomic branch and differences in the abundance of bacterial members between the two groups. The relative abundance of *Bifidobacterium*, *Bacteroides*, *Bacillus*, *Lactobacillus*, *Faecalibacterium*, and *Ruminococcus* were significantly decreased in the WP group than the SP group (Wilcoxon rank sum test). Conversely, we observed that the genera *Butyrivibrio*, *Coprococcus*, *Roseburia*, and *Succinivibrio* abundances were significantly higher in the WPs than in the SPs (Wilcoxon rank sum test). Moreover, in contrast to the SP group, the WP group was lack of several genera with relatively low proportional abundance, such as *Acinetobacter*, *Campylobacter*, *Enhydrobacter* and *Proteus.*

The SP and WP groups were mainly characterized by two co-occurrence networks with scattered nodes represented by multiple primary genera within distinct phyla. Both ecological networks were obviously parsed into five relatively consistent modules of co-occurring microbial members (**Figure 5F**). The highest degree and clustering coefficient score conceivably pointed out core community members within each ecosystem. The result showed that *Firmicutes* was the keystone species for determining the network in both the SP and WP groups, while the role of *Bacteroidetes* was minimized in the WP community (**Figure 5F** and **Supplementary Table 5**). We also undertook a detailed statistical analysis of the network characteristics including modularity, diameter, density, average clustering coefficient, and so on. A comparison was carried out for these network property parameters and node-level topological features between the two groups, the result indicated that the overall complexity and composition of the networks for the WP group were clearly reduced compared with those of the SP group (**Figures 5G, H** and **Supplementary Table 6**). Genera with larger degree centrality scores were considered as keystone taxa in the SP group, such as *Peptococcus*, *Ruminococcus*, *Bifidobacterium*, *Lactobacillus*, and *Bacteroides*; however, these genera contributed less to network construction in the WP group, within which *Anaerovibrio*, *Pyramidobacter*, *Butyrivibrio*, and *Collinsella* were more influential.

### Metabolomics Analysis Identified Different Metabolic Patterns Between the SP and WP Groups

**Figure 6:**
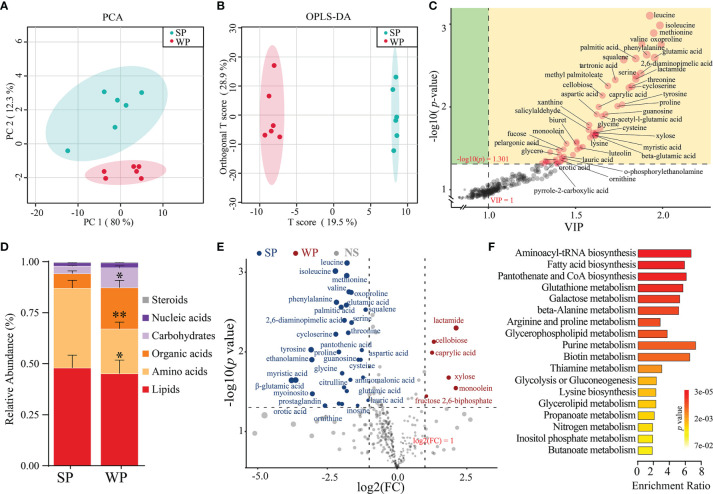
Differences in metabolic patterns between the SP and WP groups. **(A)** The principal coordinate analysis (PCA) plot revealed a separation of the metabolites between the two groups. **(B)** Orthogonal partial least squares discriminant analysis (OPLS-DA) of the metabolomics profiles in the colon. **(C)** Dot diagram determined 56 individual metabolites based on the double criteria of VIP ≥1.0 and *P* < 0.05 (Student’s *t*-test). These metabolites strongly contributed to the discrimination of the two groups. **(D)** Stacked bar graph showing the differences in relative abundance of six metabolite categories between the two groups. **(E)** Volcano plots of significantly changed metabolites in the SP and WP groups. **(F)** Metabolic pathway analysis of differentially abundant metabolites between the two groups. The *P-*value for each term is represented by the color intensity. The data are presented as means ± SEM for **(D)**, *P < 0.05, **P < 0.01, Student’s *t*-test; *n* = 6.

A total of 215 metabolites were identified (**Supplementary Table 7**). The samples from the SP and WP groups were clearly separated according to the PCA and OPLS-DA (R2Y = 0.997, Q2 = 0.829, permutation test, **Supplementary Figure 8**), which suggested that the weaning promoted the development of a metabolome composition distinct from that present in breastfeeding (**Figures 6A, B**). We identified the 56 individual metabolites that most contributed to the discrimination between the two groups based on the following criteria: a VIP score >1.0 from OPLS-DA modeling and a |log10 (*P-*value) | >1.3 (Student’s *t* test) (**Figure 6C**). We classified all the metabolites into six major categories. The differences were displayed in overall metabolic clusters between the two groups through a stacked bar graph (**Figure 6D**). We found a higher abundance of organic acids and carbohydrate-associated metabolites in the WP group than in the SP group, whereas lower abundance of amino acids in the WP group. The WP group was largely characterized by a reduction in amino acid (including leucine, isoleucine, lysine, methionine, threonine, valine, and phenylalanine) and purine (including xanthine, inosine, guanosine, and 5-aminoimidazole-4-carboxamide) metabolites (**Figure 6E**). In contrast, the abundance of 9 metabolites associated with carbohydrate and lipid metabolism was significantly increased in the WP group than in the SP group (e.g., cellobiose, octanoate, xylose, monoolein, pelargonic acid, and glycerol) (**Supplementary Table 7**). KEGG pathway analysis of these significantly changed metabolites identified 19 different enrichment pathways between the two groups (**Figure 6F**). The key enrichment pathways identified were aminoacyl-tRNA biosynthesis (enrichment ratio [ER] = 6.24), fatty acid biosynthesis (ER = 5.72), pantothenate and CoA biosynthesis (ER = 5.92), glutathione metabolism (ER = 5.43), and purine metabolism (ER = 7.89).

### Correlation Analysis and Functional Prediction of the Microbiome

**Figure 7:**
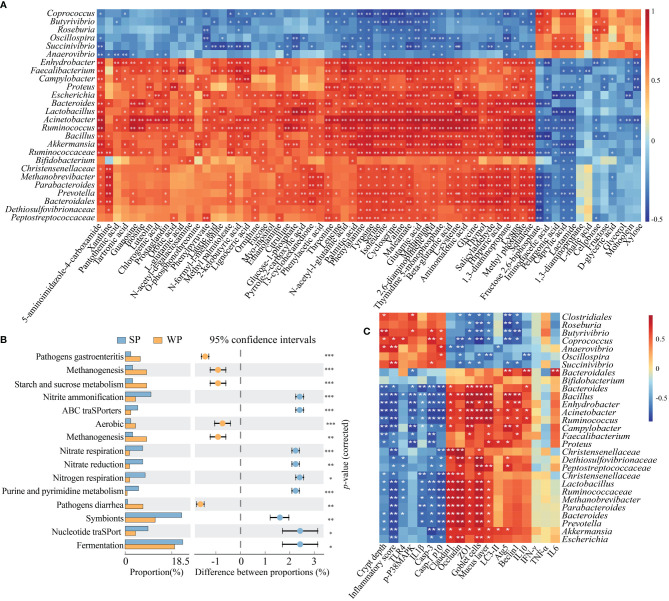
Correlation and pathway analysis of significant differentially abundant metabolites and bacteria. **(A)** Heatmap showing the correlations between gut bacterial genera and individual metabolites. Spearman’s rank correlation coefficients and *P-*values for the correlations of genera and their metabolites were calculated. The correlation effect is indicated by a color gradient from red (positive correlation) to blue (negative correlation). **(B)** The enriched pathways of microbial genes between the SP and WP groups. **(C)** Spearman’s correlation analysis between the colonic microbiota and gut barrier integrity or inflammation-related signaling parameters. *P < 0.05, **P < 0.01, ***P < 0.001.

The results of the 16S rRNA sequencing showed that early weaning disturbed the taxonomic composition of the colonic microbiota. Moreover, a metabolomics analysis indicated that the metabolic profiles of the two groups were different. We further explored the functional correlations between the 26 identified bacterial genomes and the 68 altered colon metabolites. Analysis based on Spearman’s correlation coefficient revealed 362 significant associations, many of which involved genera enriched in the SP group. For instance, within the amino acid class, seven metabolites were positively associated with the genera *Lactobacillus*, *Bacillus*, *Bacteroides*, and *Ruminococcus* (*r*≥0.621, FDR-corrected *P-*value <0.01) but negatively correlated with *Coprococcus*, *Butytivibrio*, and *Succinivibrio* (*r*≥0.489, FDR-corrected *P-*value <0.05). For purine metabolism, the genera *Ruminococcus*, *Acinetobacter*, *Lactobacillus*, and *Bacteroides* were positively correlated with inosine, guanosine, and 5-aminoimidazole-4-carboxamide (*r*≥0.585, FDR-corrected *P-*value <0.05). For carbohydrate metabolism, most genera enriched in the SP group were negatively correlated with lactamide, caprylic acid, pelargonic acid, iminodiacetic acid, and fructose 2,6-biphosphate (*r*≥0.422, FDR-corrected *P-*value <0.05) (**Figure 7A**). Next, the abundances of the original 16S rRNA sequences were processed with PICRUSt2 to impute the putative biological functional compositions of the microbiomes in the SP and WP groups (**Figure 7B**). In general, symbionts and vigorous fermentation were enriched in the SP group, while pathogens gastroenteritis- and pathogens diarrhea-related genes were more abundant in the WP group. Among these predicted metabolic functions, the abundances of functional genes associated with methanogenesis, starch and sucrose metabolism, and aerobic metabolism significantly increased in the WP group than in the SP group, which suggested that early weaning led to the disturbance of intestinal flora linked with gut inflammation and a shift in metabolic function. In contrast, we found that the gut microbiota of the SP group was characterized by significant enrichment of several metabolism-related pathways. These pathways included nitrogen respiration, nitrate reduction, nitrate respiration, nitrite ammonification, and nucleotide metabolism. Spearman’s correlation matrix suggested that the imbalance of the gut microbiota influenced mucosal inflammation and barrier integrity (**Figure 7C**). The relative abundance of *Bifidobacterium*, *Bacteroides*, *Bacillus*, *Lactobacillus*, *Faecalibacterium*, and *Ruminococcus* decreased in the WP group, which was positively correlated with the expression levels of ZO-1, occludin and claudin 3, the number of goblet cells, and mucosal layer thickness. In contrast, the increased relative abundance of *Butyrivibrio*, *Coprococcus*, and *Succinivibrio* in the WP group was positively associated with crypt depth and inflammation scores.

## Discussion

The demand for efficient and healthful pig production has posed challenges for the implementation of early weaning in the modern swine industry (34). Multiple stressors encountered during the early weaning contribute to transient anorexia, gut inflammation, and the disturbance of the intestinal flora of piglets (2). The exploration of cross-interaction between GI inflammation, gut microbiota and metabolomic in weaning piglets is prerequisite of develop strategies to overcome weaning stress and optimize the weaning transition (35). Our integrated analysis revealed that early weaning caused serious colonic inflammation in the piglets, which is linked with impaired mucosal barrier function. We also found that autophagy and apoptosis-related signaling pathways played an important role in promoting the inflammatory process. TLR4 is a major inducer of apoptosis. We observed high levels of TLR4 activation in the WP group, which suggested that the occurrence of intestinal inflammation post-weaning was related with the disturbance of the gut microbiome (13). Although there was a partial overlap in the composition of the gut microbiome and metabolome profiles between the SP and WP groups, the colonic symptom-associated features identified in the current study presented differences between the two groups.

Consistent with previously reported results, weaning stress resulted in severe damage to the colon, which morphologically manifested as high crypt density loss, hyperplasia, and compromised mucosal integrity (36, 37). The intestine of weaning piglets is not fully functionally developed and cannot adapt to sudden dietary transitions and/or environmental changes, which may be the main cause of weaning-associated intestinal inflammation. Impaired barrier function leads to excessive leakage of intraluminal antigens, toxins, and the consequent activation of an intestinal immune response (38). Indeed, the loss of the gut mucus layer and the reduction in the levels of tight junction proteins were evident in the piglets after weaning. Deficiencies in these components indicated that the capacity of the intestinal barrier to defend against pathogens and luminal antigens was impaired (39). These observations suggested that an imbalance in autophagy and apoptosis resulting from weaning stressors could be involved in the etiology of post-weaning enteric inflammation.

In the current study, the TUNEL and TEM analyses confirmed that discontinuous mucosal and transmural inflammation was the noticeable worsening of cell apoptosis seen in the colon tissue of weaned piglets. Meanwhile, ELISA analysis showed that the relatively higher production of pro-inflammatory cytokines (IL-1β, IL-6, and TNF-α) were in the WP group than in the SP group, which indicated that early weaning led to the systemic inflammatory cytokine profiles of the piglets. Conversely, the WP group showed a reduced IL-10, which often exerts critical suppressive effects on the progress of gut inflammation (40). Changes in the release levels of these cytokines can modulate autophagy and apoptosis in response to adverse conditions, such as dysbiosis of the gut microbiota (41). Notably, we found that the level of the autophagosome marker LC3 was reduced in colon tissues of the weaned piglets, suggesting a deficiency in autophagosome elongation and assembly. Beclin-1 and ATG5 proteins are critical for the completion of autophagy involving the LC3-conjugation machinery (42, 43). These autophagy-related molecules were markedly downregulated in WPs, which may account for the decreased autophagic flux seen in the colon of the weaned piglets. Our results supported that a link exists between autophagy and goblet cell function, which is in line with a previous report (44). The emerging evidence has indicated that appropriate levels of autophagy in the intestinal epithelium functions as a deterrent to cytokine-meditated programmed cell apoptosis, thereby promoting intestinal homeostasis and limiting immunopathology (43, 45). In the current study, autophagy deficiency and an abnormal pro-inflammatory cytokine elevation occurred simultaneously in the WP group, which suggested that weaned piglets are susceptible to immunopathology resulting from the activity of proinflammatory cytokines, particularly IL-1β. Accordingly, we further tested the release and activation status of IL-1β. TLR4 modulates the secretion of various cytokines following the sensing of exogenous pathogen-associated molecules, which plays a significant role in mediating gut microbiota disorder-induced enteric infections (46). Similarly, we found that TLR4 and P38MAPK were highly expressed, and P38MAPK phosphorylation was also enhanced in the WP group. Upon microbial invasion, the activation of TLR4/P38MAPK signaling accelerates P38MAPK phosphorylation, which further induces the release of IL-1β (14, 47). The expression of caspase-1 p10 was also markedly higher in the WP group than in the SP group. Caspase-1 p10 is an auto-cleaved fragment of caspase-1 responsible for cleaving pro-IL-1β into mature form to induce cell death (48). Additionally, the key executor of the apoptotic process, caspase-3, was also highly activated in the WP group, which confirmed the occurrence of severe apoptotic progression during early weaning. Collectively, these results suggested that early weaning led to reduced autophagy and aberrant elevated apoptosis in the colon.

Early weaning caused a reduction in gut microbial richness and diversity and shifted the microbial community in piglets, which was in line with previous findings (49, 50). This reduction in gut microbial richness and diversity might be due to the imbalance between symbionts and potentially pathogenic microorganisms by assessment of PICRUSt2 in this study. Differences in sequence abundance in each taxonomic branch were indicative of the existence of taxonomic perturbations, which allowed for distinguishing the WP group from the SP group. *Bifidobacterium*, *Bacillus*, *Bacteroides*, and *Lactobacillus* were abundant in the SP group than in the WP. Most of these bacteria are considered as probiotics for a long history (51, 52). *Faecalibacterium* was found to exert anti-inflammatory effects on both cell and mouse models of colitis (53). The absence of these commensal bacteria may be relative with the gut physiology dysbiosis of the piglets in the WP group. Meanwhile, the gut microbial ecosystem in WPs was characterized by the enrichment of *Butyrivibrio* and *Succinivibrio*. The Spearmen correlation analysis suggested that the differentially abundant gut microbiota community of the WP group was significantly associated with intestinal barrier function and inflammation-related signals. Such disturbances in the intestinal flora and loss of microbial biomass at the early stage of life have previously been reported to be highly associated with GI diseases (54).

Major shifts in microbial composition will lead to an unstable network of microbes, which is often associated with poor host health (55). In this study, co-occurrence analysis was employed to discern the differences in network associations across microbial communities from the SP and WP groups. The bacterial interactions were more intricate and extensive in the SPs than in the WPs. Empirical work has shown that reduced ecological interaction frequencies can disrupt the stability of the microbiome within the mammalian intestine, with detrimental consequences for the health of the host (55). Moreover, based on node properties, we found that the importance of some community members in the network had changed in the weaned piglets. Although five relatively consistent modules of co-occurring gut microbes were detected in both the SP and WP groups. Some species with high ‘degree’ and ‘clustering coefficient’ scores contributed equally to the networks of the SP group, which were evenly distributed and dispersed. In contrast, several important species played a dominant role in constructing the network of the WP group, such as *Anaerovibrio*, *Roseburia*, *Pyramidobacter*, *Butyrivibrio*, and *Collinsella.* In addition to changes in diversity, symbiotic relationships tended to be concentrated and monotonous in the WP group, which would be expected to exert a long-term adverse effect on the intestinal health of the piglets (56–58).

The gut metabolites can mediate the interactions between the gut microbiota and the host (16, 21). Multiple-omics analyses yielded unique and differential features of microbiota-metabolic correlations between the SP and WP groups. The abundances of leucine, isoleucine, and lysine were significantly decreased in the WP group than in the SP group. These metabolites were positively correlated with the number of dominant bacteria in the SP group based on correlation analysis. Meanwhile, the prediction of microbial biological function showed that the pattern amino acid metabolites utilization mediated by the intestinal microflora had collapsed in the weaned piglets. The loss of amino acid metabolites may cause an aberrant progression of mucus synthesis (59), because the synthesis of ‘mucin domain’, a core element of mucins, requires various amino acid residues (60). Furthermore, early weaning resulted in a prominent decrease in the levels of purine metabolites such as xanthine, inosine, and guanosine. We also found that several bacteria, such as *Bacillus*, *Escherichia*, *Akkermansia*, and *Ruminococcus*, may be involved in purine metabolism, which is consistent with previous studies (61, 62). A previous study reported that gut microbial-derived purine metabolites could attenuate intestinal barrier dysfunction (62). These findings may partly explain the inflammatory response present in the colon of the weaned piglets.

In conclusion, early weaning resulted in damage to colonic crypt architecture and a severe inflammatory response, which may be attributable to the dysregulation of autophagy and the TLR4/P38MAPK/IL-1β-mediated apoptotic signaling pathway. Meanwhile, early weaning not only caused the loss of richness and diversity of the microbial community and the dysregulation of the pattern of co-occurrence, but also concomitantly induced metabolic changes in the colonic content. Gut microbiota disturbance caused by early weaning inhibited autophagy and activated the TLR4/P38MAPK/IL-1β apoptosis signaling pathway, ultimately aggravated colon inflammation. These findings provide new knowledge concerning exploiting microbial metabolites to regulate gut barrier integrity and immune responses in weaned piglets.

## Data Availability Statement

The datasets generated and analyzed during the current study are available from the corresponding author on reasonable request. The raw sequencing data are available from the NCBI SRA database under accession number PRJNA761570 (https://www.ncbi.nlm.nih.gov/sra/PRJNA761570).

## Ethics Statement

The animal study was reviewed and approved by This research was specifically approved by the Animal Care and Use Committee of Zhejiang University (Ethics Code Permit ZJU20170529).

## Author Contributions

HW and WT conceived the study. HW and WT designed the project and the experiments. WT, JLL, YM, and HW collected the samples, prepared the 16S rRNA library for sequencing, and performed the experiments. WT and YW performed the bioinformatics analysis. JXL, JLL, and YM helped with IF staining. WT wrote the manuscript. HW, JXL, YM, JLL, and YW revised the manuscript. All the authors reviewed and approved the final version of the manuscript.

## Funding

This study was supported by grants from the Key R & D Projects of Zhejiang Province (2022C04034), the National Natural Sciences Foundation of China (31672430), the Natural Science Foundation of Zhejiang Province (Z19C170001), the Funds of Ten Thousand People Plan of China, and the National Key Research and Development Program of China (2017YFD0500502).

## Conflict of Interest

The authors declare that the research was conducted in the absence of any commercial or financial relationships that could be construed as a potential conflict of interest.

The handling Editor declared a past co-authorship with one of the authors HW.

## Publisher’s Note

All claims expressed in this article are solely those of the authors and do not necessarily represent those of their affiliated organizations, or those of the publisher, the editors and the reviewers. Any product that may be evaluated in this article, or claim that may be made by its manufacturer, is not guaranteed or endorsed by the publisher.
