# Supplementary material for: Impairment of Intestinal Barrier Function Induced by Early Weaning via Autophagy and Apoptosis Associated With Gut Microbiome and Metabolites
Source: Front Immunol. 2021 Dec 15;12:804870. doi: 10.3389/fimmu.2021.804870 (PMC8714829; doi:10.3389/fimmu.2021.804870)
Supplement: Supplementary file 1 [file DataSheet_1.docx]

Supplementary Material

# Supplementary Figures and Tables

## Supplementary Figures

##
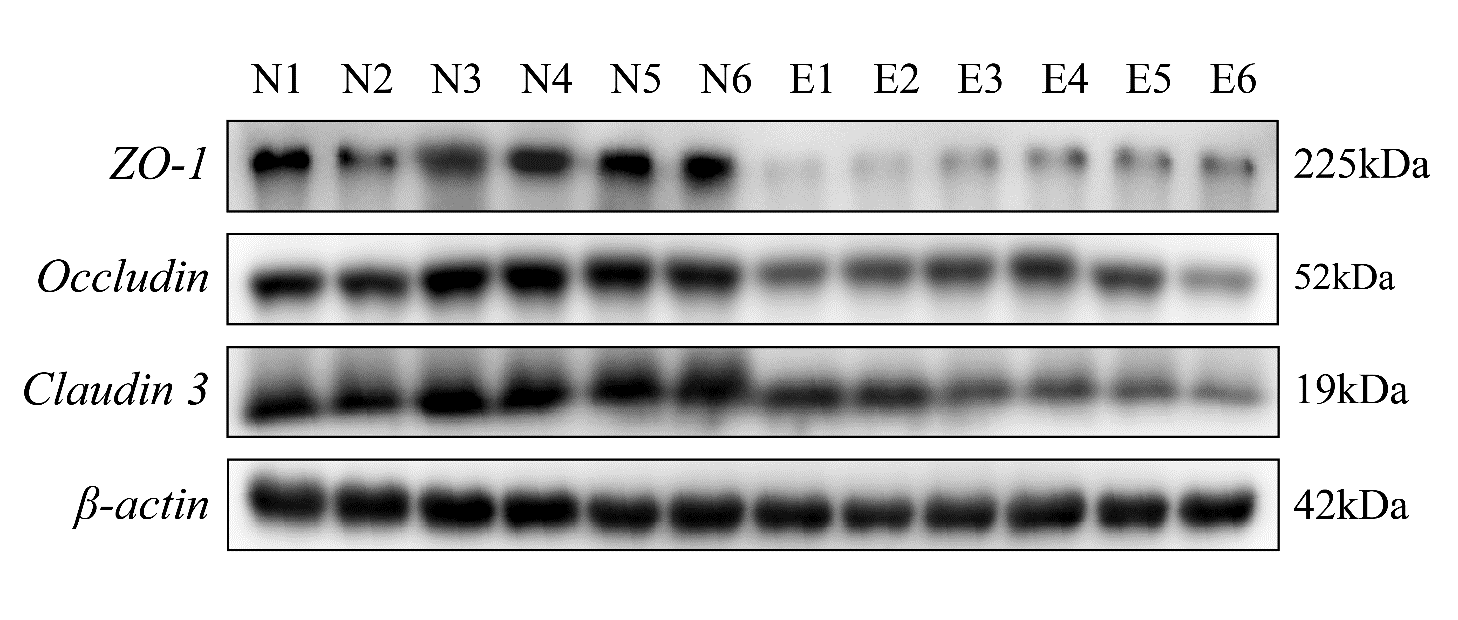
Supplementary Figure 1. Uncropped blots images of barrier function related protein form the overall sample were shown.

**
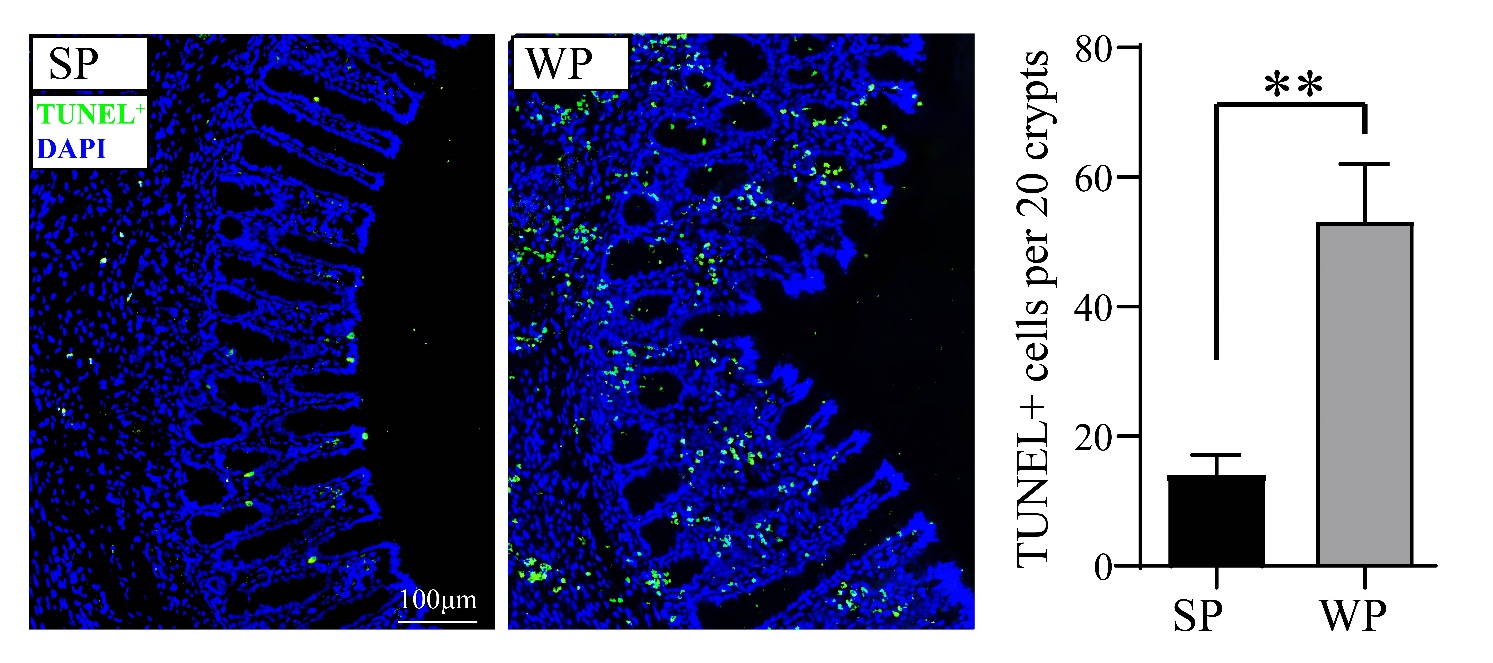
**

**Supplementary Figure 2.** Representative images of TUNEL-labelled colonic epithelium (green) and statistical analysis of TUNEL positive cell count per 20 crypts. Scale bars, 100 μm. Student’s t test; error bars: SEM; n = 6.

**
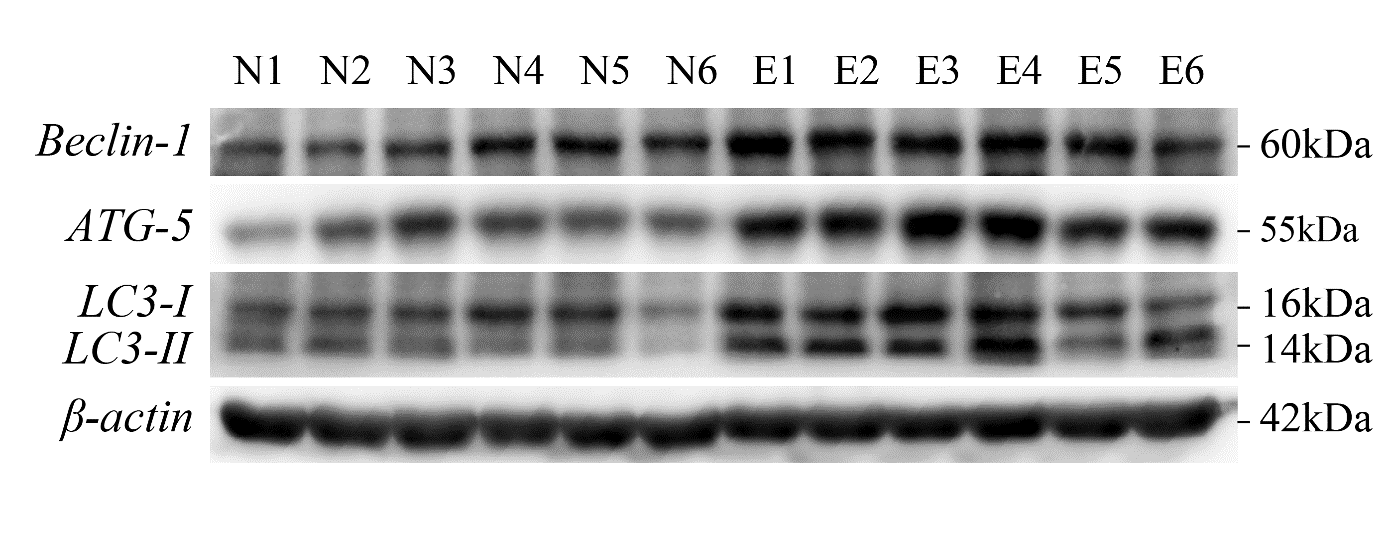
**

**Supplementary Figure 3.** Uncropped blots images of autophagy related protein form the overall sample were show

**
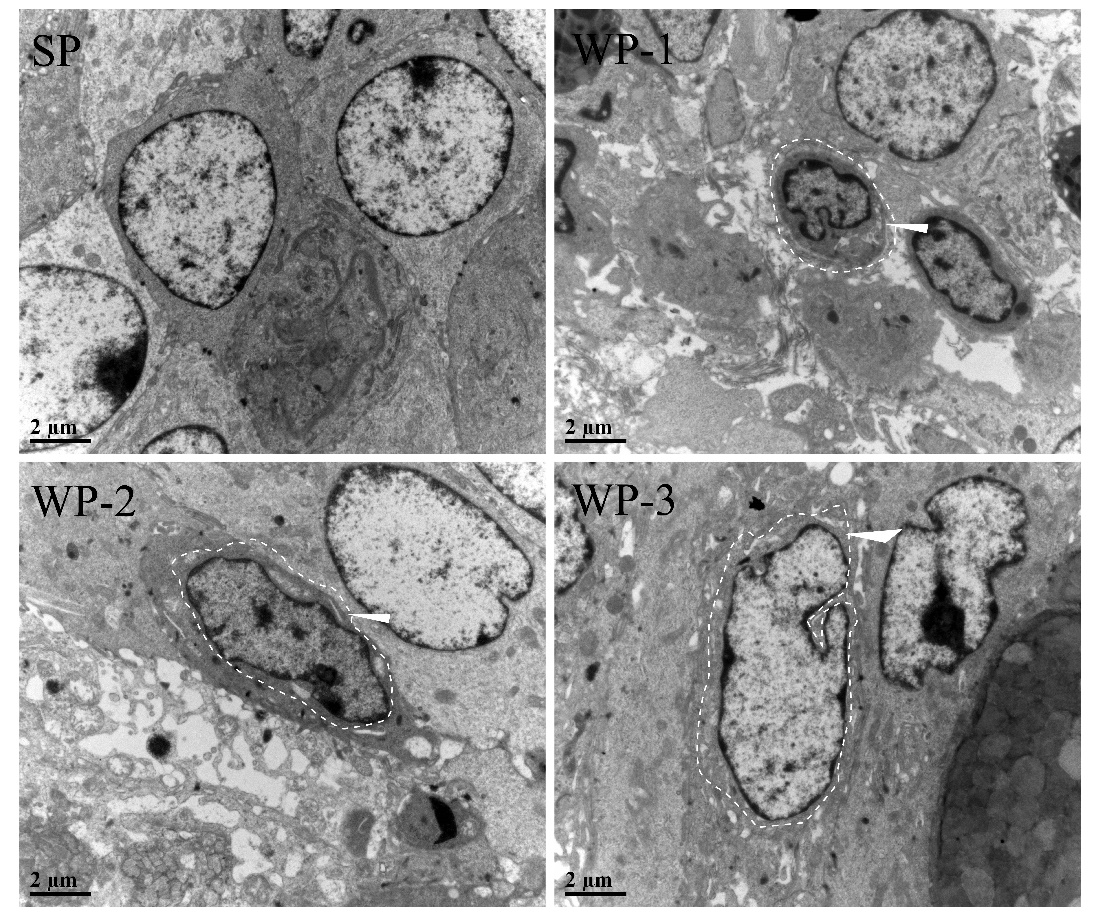
**

**Supplementary Figure 4.** Higher magnification (scale bars, 2μm. ×10,000) of transmission electron microscopy images of colonic crypts from SP and WP (1-3). Multiple electron microscope fields from WP group demonstrated typical apoptosis phenomenon, characterized with broken nuclei and leak of nuclear mass.

**
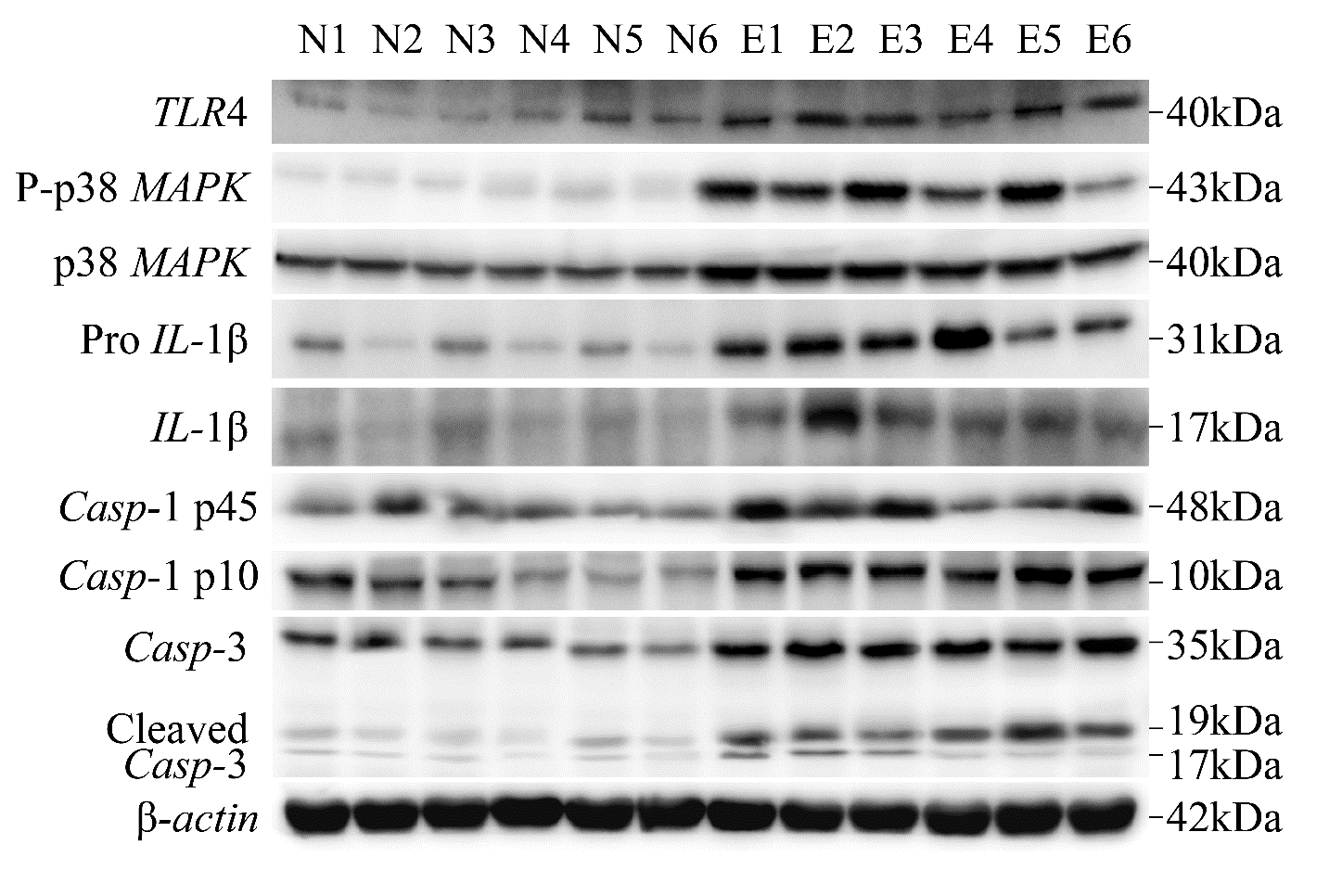
**

**Supplementary Figure 5.** Uncropped blots images of apoptosis related protein form the overall sample were shown.

**
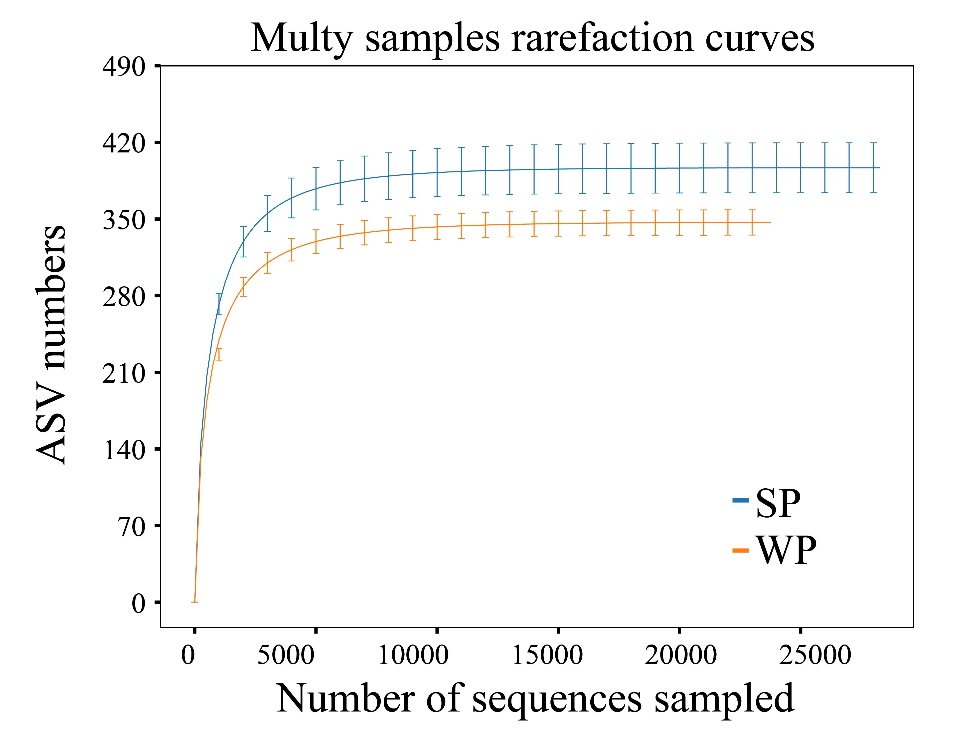
**

**Supplementary Figure 6.** Rarefaction Curve. X-axis: Counts of randomly sampled sequences; Y-axis: Counts of features detected by giving sequences. Lines with different colors stand for different groups.

**
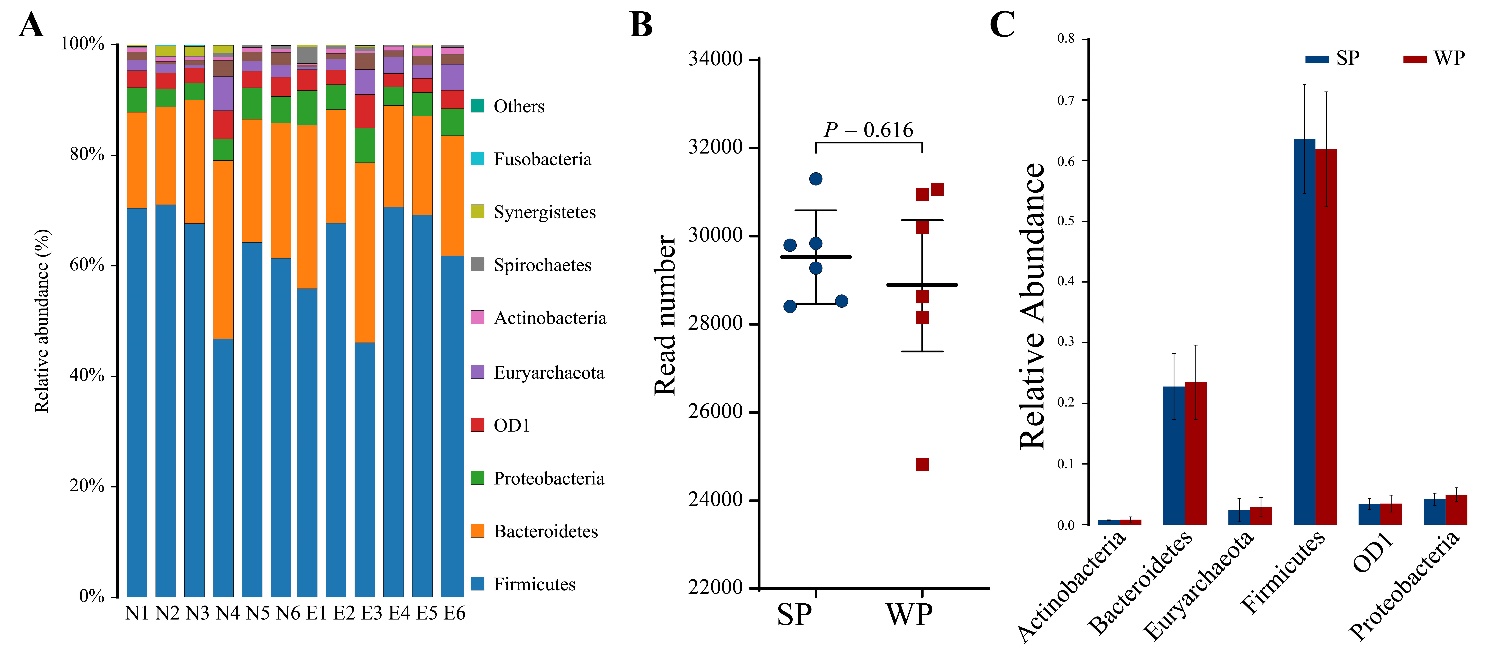
**

**Supplementary Figure 7.** Species annotation and taxonomic analysis at phylum level. (**A**) Histogram of species distribution at phylum level from each sample. (**B**) Statistics of reads count in phylum level. Student’s t test; error bars: SEM; n = 6. (**C**) Histogram of phylum level rank sum test analysis. Wilcoxon rank-sum test; n = 6.

**
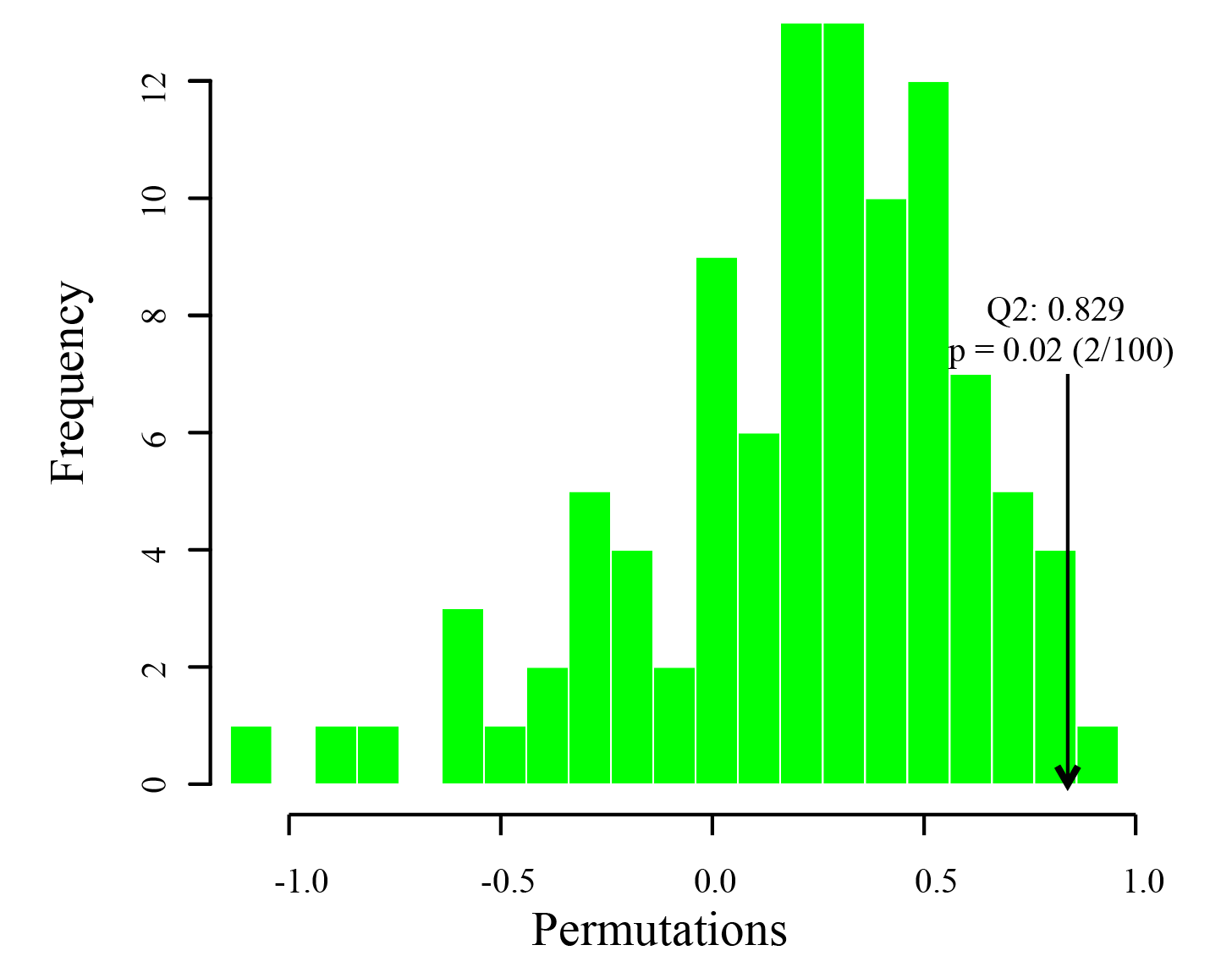
**

**Supplementary Figure 8.** Permutation test diagram for OPLS-DA model validation. X-axis represents the accuracy of the model. The Y-axis represents the frequency of accuracy of 200 models in 200 permutation tests. The arrow represents the location of model accuracy. Q2 represents the predictive power of the mode.

# Supplementary Tables

**Supplementary Table 1.** **Histological damage score**

| Score | Inflammation extent | Bleeding | Crypt hyperplasia | Crypt density loss |
| --- | --- | --- | --- | --- |
| 0 | None | None | None | None |
| 1 | Mucosa | Mild | Mild | Basal 1/3 damage |
| 2 | Submucosa | Moderate | Moderate | Basal 2/3 damage |
| 3 | Transmural | Severe | Severe | Crypt lost |

**Supplementary Table 2.** **Primary antibody information.**

| Antibody | Source | Identifier | Dilution |
| --- | --- | --- | --- |
| ZO-1 | Invitrogen, Camarillo, CA, USA | 33-9100 | 1:1,000 |
| Occludin | Invitrogen, Camarillo, CA, USA | 33-1500 | 1:1,000 |
| Claudin 3 | ABCAM, Cambridge, UK | ab214487 | 1:1,000 |
| β-actin | CST, Danvers, MA, USA | 13E5 rabbit mAb #4970 | 1: 800 |
| Beclin-1 | CST, Danvers, MA, USA | D40C5 Rabbit mAb #3495 | 1: 800 |
| ATG5 | CST, Danvers, MA, USA | D5F5U rabbit mAb #12994 | 1: 800 |
| LC3A/B | CST, Danvers, MA, USA | D3U4C XP rabbit mAb #12741 | 1: 800 |
| Caspase-1 | CST, Danvers, MA, USA | E2Z1C rabbit mAb #24232 | 1:1,000 |
| Caspase-3 | CST, Danvers, MA, USA | #9662 | 1:1,000 |
| P38MAPK | CST, Danvers, MA, USA | D13E1 XP rabbit mAb #8690 | 1:1,000 |
| (p)-P38 MAPK | CST, Danvers, MA, USA | Tyr182 rabbit mAb #4511 | 1:1,000 |
| TLR4 | CST, Danvers, MA, USA | D8L5W rabbit mAb #14358 | 1:1,000 |
| IL-1β | CST, Danvers, MA, USA | D6D6T rabbit mAb #31202 | 1:1,000 |
| MUC2 | Biorbyt, Cambridge, UK | rabbit anti-MUC2 orb372331 | 1: 400 |

**Supplementary Table 3.** **Information of raw data quality control**

| Sample ID | Raw Reads | Clean Reads | Denoised Reads | Non-chimeric Reads | The Good' coverage |
| --- | --- | --- | --- | --- | --- |
| SP1 | 38596 | 35383 | 32174 | 30685 | 0.9951 |
| SP2 | 34791 | 31902 | 29858 | 29270 | 0.9963 |
| SP3 | 38149 | 36756 | 34034 | 31192 | 0.9854 |
| SP4 | 34449 | 31440 | 29480 | 29270 | 0.9949 |
| SP5 | 36512 | 33978 | 33099 | 32694 | 0.9910 |
| SP6 | 34818 | 32007 | 30901 | 30374 | 0.9956 |
| WP1 | 38452 | 34966 | 31449 | 29596 | 0.9983 |
| WP2 | 37279 | 33906 | 32659 | 31721 | 0.9952 |
| WP3 | 33638 | 30713 | 29521 | 28832 | 0.9954 |
| WP4 | 33721 | 32387 | 29409 | 25022 | 0.9915 |
| WP5 | 36949 | 34150 | 32882 | 32017 | 0.9948 |
| WP6 | 37271 | 34278 | 31800 | 30896 | 0.9948 |

**Supplementary Table 4.** **Alpha diversity metrics**

| Group | SP | WP | *P* value |
| --- | --- | --- | --- |
| Feature | 399.17±21.17 | 342.16±9.96 | 0.0920 |
| Shannon | 6.89±0.11 | 6.52±0.05 | 0.0150 |
| Simpson | 0.98±0.003 | 0.97±0.001 | 0.0650 |
| Chao1 | 422.74±32.35 | 354.55±10.78 | 0.1800 |
| PD_whole_tree | 19.70±1.79 | 17.45±0.70 | 0.025 |

**Supplementary Table 5. Node properties describing its situation in the network.** (using separate Excel file)

**Supplementary Table 6.** **Comparison of network characterization between the two groups**

| Network properties | SP | WP |
| --- | --- | --- |
| Number of nodes | 52 | 46 |
| Number of edges | 188 | 98 |
| Modularity | 0.565 | 0.533 |
| Number of communities | 5 | 5 |
| Network diameter | 6 | 8 |
| Network Density | 0.118 | 0.095 |
| Average shortest path length | 2.405 | 3.823 |
| Average clustering coefficient | 0.631 | 0.514 |

**Supplementary Table 7. Comparison of metabolite composition between the two groups.** (using separate Excel file)

**Supplementary Table 8. List of 9 significantly increased metabolites in the WP group and 52 significantly increased metabolites in the SP group.** (using separate Excel file)
